# Porous Gold: A New Frontier for Enzyme-Based Electrodes

**DOI:** 10.3390/nano10040722

**Published:** 2020-04-10

**Authors:** Paolo Bollella

**Affiliations:** Department of Chemistry and Biomolecular Science, Clarkson University, Potsdam, NY 13699-5810, USA; pbollell@clarkson.edu

**Keywords:** porous gold (PG), modified electrodes, enzyme-based biosensors, enzyme fuel cells (EFCs), bioelectrocatalysis

## Abstract

Porous gold (PG) layers modified electrodes have emerged as valuable enzyme support to realize multiple enzyme-based bioelectrochemical devices like biosensors, enzymatic fuel cells (EFCs), smart drug delivery devices triggered by enzyme catalyzed reactions, etc. PG films can be synthesized by using different methods such as dealloying, electrochemical (e.g., templated electrochemical deposition, self-templated electrochemical deposition, etc.) self-assembly and sputter deposition. This review aims to summarize the recent findings about PG synthesis and electrosynthesis, its characterization and application for enzyme-based electrodes used for biosensors and enzymatic fuel cells (EFCs) development.

## 1. Introduction

**Figure 1:**
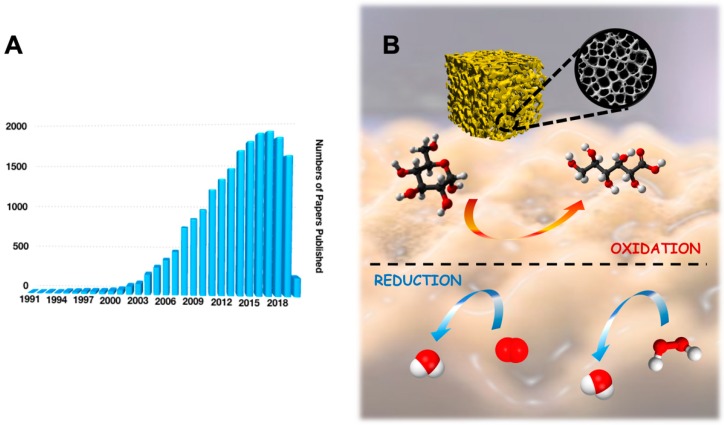
(**A**) Statistical data for number of papers published in the last 30 years by searching ‘gold nanostructures’ in Web of Science on 1 March 2020. (**B**) Porous gold structure that is able to catalyze a wide range of reactions at the electrode surface (e.g., glucose oxidation, oxygen reduction, hydrogen peroxide reduction, etc.).

In the last decades, upgrading the features and performance of electrocatalysts has been one of the most important topics in electrochemistry addressing a particular attention towards improving the electron transfer (ET) reaction of redox enzymes as well as the enzyme loading for biosensing and enzymatic fuel cells (EFCs) purpose [1,2]. Among all the catalysts, the use of gold and its nanostructures have tremendously increased in the last 15 years, which has been reported as a new gold “rush” era [3,4]. Hundreds of papers about gold and its nanostructure are published annually, as shown in Figure 1A (data extrapolated from Web of Science by looking for ‘gold nanostructures’). According to tradition, gold in its bulk form had always been considered as an inert/inactive metal [5]. However, the discovery that gold nanostructures can act as catalysts for heterogeneous reactions in both gas and liquid phases, triggered the spread of gold usage in a wide range of catalytic reactions, as shown in Figure 1B [6,7,8].

Gold nanostructures can be synthesized by using either top-down methods (slicing or successive cutting of bulk materials till nano sized particles) such as wet synthesis (e.g., chemical reduction, green synthesis, etc.) and electrosynthesis or bottom up methods (gold atoms tend to aggregate or self-assemble till forming clusters) such as mechanical milling, etching, laser ablation, sputtering and electro-explosion [9,10,11,12]. Notably, many gold nanostructures have been obtained by using bottom up process (e.g., spheres, rods, stars, cubes, etc.) [13]. These nanostructures exhibit peculiar chemical–physical properties and their external shell modified with several functional groups (e.g., –COOH, –NH_2_, –OH, etc.) are responding to environmental changes like temperature, pH, light and redox potentials [14,15,16]. In this regard, to avoid gold nanoparticles coalescence and preserve their properties for applicative purposes, gold nanoparticles are usually functionalized or capped with appropriate organic molecules [17]. In particular, the capping agent plays a dual role: (i) protecting and stabilizing gold nanoparticles, preventing their aggregation; (ii) making a hybrid system (gold nanoparticles capped with organic linkers) that suits better for some specific biomedical applications [18]. Beyond the gold nanoparticles chemical modification, a possible way to improve the catalytic properties of gold nanoparticles is the increasing of their surface area. Commonly, a larger surface area can be achieved by preparing nanoparticles with a rough or porous surface [19]. These kinds of morphological features have a strong impact on the overall behavior of nanoparticles (chemical–physical properties) [20].

Besides gold nanoparticles as such or deposited onto the electrode surface, porous gold (PG) films modified electrodes have attracted an increased interest of electrochemical community due to the extraordinary enlargement of surface area that allows one to increase the enzyme loading and the ET features of immobilized redox enzymes.

Porous gold (PG) can be defined as a gold micro-/nanostructure with a regular porous structure exhibiting an extremely high surface-to-volume ratio. PG can be classified based on the pore size according to International Union of Pure and Applied Chemistry (IUPAC) definition given for a porous nanomaterial: 0.2–2 nm pores (microporous nanomaterial), 2–50 nm (mesoporous nanomaterial) and 50–1000 nm (macroporous nanomaterial) [5,21]. However, PG films can be synthesized by using different methods such as dealloying, electrochemical (e.g., templated electrochemical deposition, self-templated electrochemical deposition, etc.) self-assembly and sputter deposition [22,23,24].

Although PG layers modified electrodes showed interesting electrochemical features, also the synthesis of porous gold nanoparticles has been recently explored in order to obtain a new zero-dimensional nanomaterial with better catalytic properties compared to two-dimensional nanomaterial like PG films [19]. In fact, porous gold nanoparticles exhibit extraordinary electrocatalytic activity and stronger plasmon resonance compared to smooth gold nanoparticles [20]. In some cases, plasmon resonance has been reported to be correlated with the porosity of nanoparticles [25]. Notably, porous gold nanoparticles have been synthesized following protocols that were previously listed for PG films [26]. Among these strategies, the dealloying of bulk gold-based alloys is the most commonly used method. For example, with the silver/gold alloy as a substrate, porous netlike gold has been constructed through the selective dissolution of silver in a nitric acid solution. Furthermore, other synthesis methods have been investigated such as laser ablation, electrophoretic deposition, hydrothermal method, etc. [27].

Nevertheless, this review aims to summarize the recent findings about PG films synthesis and electrosynthesis, their characterization and application for enzyme-based biosensors and fuel cells, focusing its attention on the relevant results achieved with the self-templated electrodeposition methods.

## 2. Synthesis of Porous Gold (PG) Layers Modified Electrodes

As reported above, PG electrodes can be prepared by using different methods, which will be discussed in detail pointing out the advantages and drawbacks of each method.

### 2.1. Electrochemical and Chemical Dealloying

Dealloying is a corrosion process in which an alloy is physically separated through the selective dissolution of the most electrochemically active alloy component. Thus, a porous sponge composed mainly of the more noble alloy constituent forms. In particular, Erlebacher and his coworkers proposed a model for the gold–silver (Au–Ag) dealloying process highlighting that the porosity is mainly due to an intrinsic dynamical pattern formation process [28]. Therefore, the pores are formed because of the chemical driven aggregation of gold atoms by a phase separation process [29].

**Figure 2:**
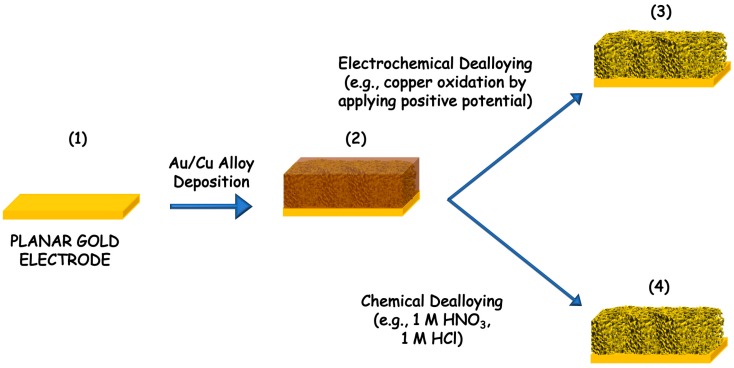
Scheme of gold dealloying process: (**1**) cleaning of planar gold electrode, (**2**) sputtering of gold/copper (Au/Cu) alloy that can be dealloyed either by (**3**) electrochemical dissolution of copper through its oxidation at positive potential or by (**4**) chemical dissolution performed in strong concentrated acids or bases solutions.

The dealloying process can be performed by using two different pathways, namely electrochemical dealloying and chemical dealloying, as shown in Figure 2. In the first, the order of dissolution is based on the electrochemical activities of the metals in the alloy. In this series, gold is the hardest metal that can be dissolved electrochemically. However, this dealloying method is not used anymore, but it is still possible to find examples in the literature about the Au-Ag alloy, Au-Zn alloy and Au-Li [30,31,32]. The porous structures can be tuned based on the ratio of the alloying metals [28]. Conversely the chemical dealloying, also called chemical etching, is based on the chemical activity of metals. In particular, gold is chemically stable in all acids or bases while silver (Ag), aluminum (Al) and copper (Cu) can be easily dissolved [26,33,34]. Moreover, the morphology of the precursor is affecting the dealloyed PG form (e.g., nanoparticles, nanowires, microwires and films). In both dealloying methods the main advantage would be the possibility to control the pore size, to have homogeneous surface (notably all pores have the same size), while the main drawbacks are the harsh conditions needed for the chemical dealloying (e.g., nitric acid HNO_3_, sulfuric acid H_2_SO_4_ or sodium hydroxide NaOH etc.) or the influence of the electrolyte composition in the electrochemical dealloying methods [35].

### 2.2. Electrochemical Deposition

Unlike the electrochemical dealloying method, the electrochemical deposition methods are not including any dealloying processes [36]. Different approaches to achieve electrochemical depositions of porous gold have been reported. In particular, the electrochemical deposition methods can be classified in two groups: templated electrodeposition and self-templated electrodeposition [37,38,39].

Deng et al. reported on the development of an electrodeposition method where a gold substrate undergoes first active electrodissolution under a diffusion control in HCl, forming AuCl_2_^−^; which is immediately disproportionated in its Au atoms that are later aggregating and depositing on the gold substrate, leading to porous gold film formation [22]. Another widely recognized electrodeposition method is the application of a fixed reduction potential, which may vary based on the supporting electrolyte and the electrode support. Moreover, the electrodeposition of gold can be obtained by depositing a layer of anodized gold. The anodization can be performed applying a highly positive potential like 10 V vs. Saturated Calomel Electrode (SCE) or even stepping different positive potential from 4 to 5 V vs. SCE for 3 min [40,41,42]. The oxidation reaction creates a salmon pink layer of anodized gold, which turns to black after the reduction, confirming the formation of a porous gold film [43,44].

In the templated electrodeposition method [45,46], PG is electrodeposited by combining the formation of a template that can be removed afterwards, with the reduction of gold at the electrode surface with the application of a fixed potential [47]. Generally, the templates can be a layer of assembled particles, biological templates, ion etched substrates, polystyrene latex and silica particles [48,49].

**Figure 3:**
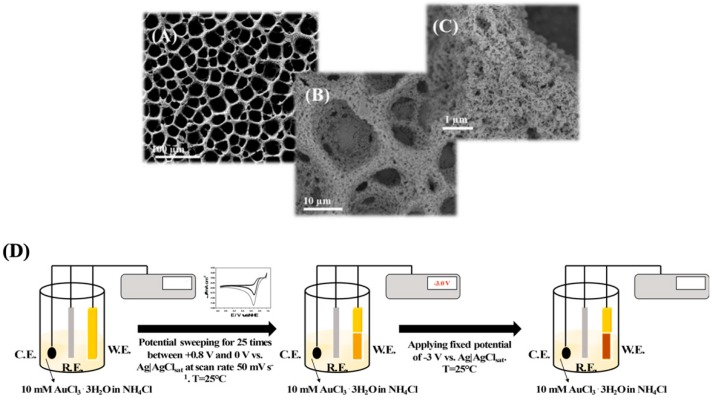
SEM images at different magnifications (**A**) 100 μm, (**B**) 10 μm and (**C**) 1 μm of the highly PG electrode obtained through a two-step electrodeposition protocol. (**D**) Scheme of the electrodeposition protocol: (i) electrodeposition of additional gold layer by using cyclic voltammetry for 25 cycles between +0.8 and 0 V vs. SCE; (ii) stepping of −3 V vs. SCE ensuring an intense hydrogen bubbling. Reproduced with permission from [52]. Copyright American Chemical Society, 2018.

However, in a self-templated electrodeposition H_2_ bubbles are generated in a solution containing a supporting electrolyte sourcing H^+^ by applying at least −2 V vs. SCE, allowing gold reduction from AuCl_4_^−^ in the interstitial space. In particular, du Toit et al. reported an electrodeposition method based on two steps: (i) the working potential was set to −0.7 V vs. SCE for 5 s creating an uneven gold surface for the adhesion of the highly porous gold (hPG) film; (ii) the potential was further stepped down to −4.0 V (vs SCE) for 10 s, which ensures an intense hydrogen bubbling, being the key for the final foam-shape of the electrode [50]. Furthermore, Sanzò et al. reported a one-step electrodeposition method by applying −3 V vs. SCE ensuring an intense hydrogen bubbling [51]. Finally, in a previous paper, we reported a two steps electrodeposition method: (i) electrodeposition of additional gold layer by using cyclic voltammetry for 25 cycles between +0.8 and 0 V vs. SCE; (ii) stepping of −3 V vs. SCE ensuring an intense hydrogen bubbling [52]. The main difference between the first two methods and the latter listed above is higher stability of the PG and its homogeneous porosity, as shown in Figure 3.

### 2.3. Self-Assembly

Self-assembly does not use any template for the electrodeposition but relies on the self-assembly process of the gold nanoparticles to form the porous structure [53,54]. Thus, the aggregation and coalescence of gold nanoparticles leads to the formation of PG. Zhang and his coworkers produced PG films from colloidal gold solution through a novel evaporation self-assembly method [54,55]. The PG films could be easily prepared by placing the gold nanoparticle solution onto the electrode letting water evaporate in standard conditions (room temperature and ambient pressure). Firstly, the gold nanoparticles are concentrated at the capillary meniscus coalescing into nanowire gold nanostructures. Different morphologies can be easily achieved by controlling the evaporation conditions. Notably, PG anisotropic films can be obtained by using this deposition method [55].

### 2.4. Sputter Deposition

Sputter deposition methods are reported as templating process or a combination of templating and dealloying procedures. The templates for PG deposition can be created either by UV light or by photolithography (notably, patterned templates can be easily obtained by using an optical mask) and finally removed by electrochemical etching. In particular, Wi et al. reported the formation of PG films firstly creating a template by lithography, then sputtering Au-Cu alloy on such a template, which is later dealloyed [56]. The great advantage of this method is to create PG films with a controlled multimodal porosity because the biggest pores are formed by using the templating method while the smallest pores are obtained by dealloying [57,58].

## 3. Characterization of Porous Gold (PG) Deposited onto the Electrodes

**Figure 4:**
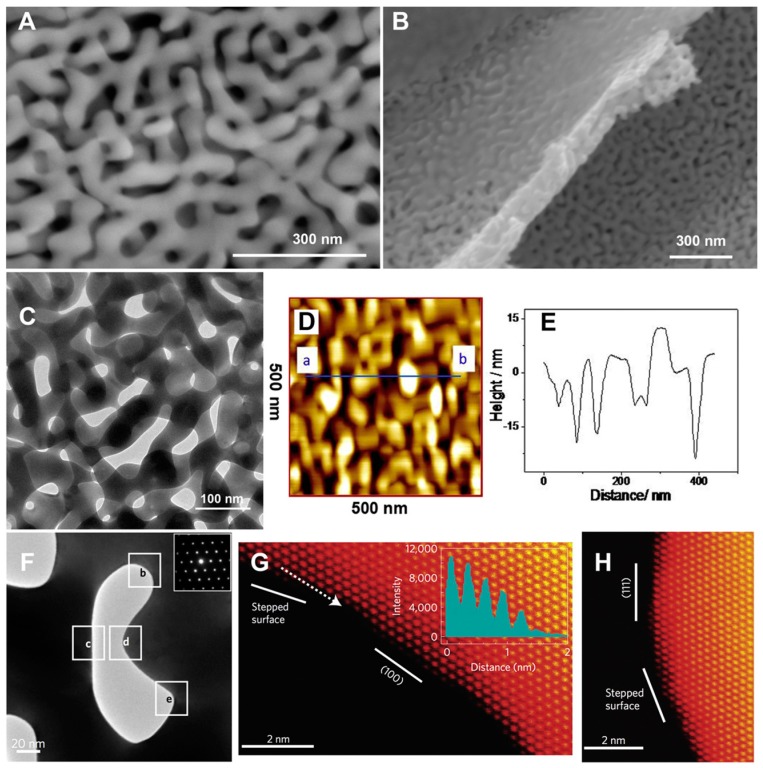
Main view (**A**) and side view (**B**) scanning electron microscope (SEM) images of a nano-porous gold (PG) leaf; (**C**) transmission electron microscope (TEM) image of nano-PG; (**D**) Contact mode atomic force microscope (AFM) images of nano-PG. Scan area: 500 × 500 nm^2^. (**E**) Height profile of a cross section along the line as indicated by a and b in (**D**); (**F**) TEM image of a [01] nanopore and the corresponding diffraction pattern (inset); (**G**) High-angle annular darkfield-scanning TEM (HAADF-STEM) image of the labeled square indicated as b in (**F**). The intensity profile (inset) along the dotted line represents a stepped surface; (**H**) HAADF-STEM image of the labeled square indicated as d in (**F**). Reproduced with permission from [60]. Copyright Elsevier Ltd., 2016.

The morphology and roughness of PG structures are critical parameters in order to improve electrochemical response of either biosensors or EFCs. Indeed, microscopy techniques like atomic force microscopy (AFM) and scanning or transmission electron microcopy (SEM/TEM) seem to be reliable imaging techniques to study the morphology of PG layers, as shown in Figure 4A–H [59,60]. TEM is particularly useful for the characterization of PG surface modification (e.g., with nanoparticles or thin films) [61,62,63]. Furthermore, TEM has been extensively used to assess different nanomaterials or conducting polymers deposited onto PG electrodes as reported in the literature [64,65]. The immobilization of immunoglobulin G (IgG) and bovine serum albumin (BSA) onto PG has been studied by using AFM, detecting individual protein molecules immobilized onto the electrode [66].

Furthermore, electrochemical techniques can be used to get deeper characterization of the electrode surface [67,68]. For example, cyclic voltammetry (CV) can be used to measure the electroactive surface areas by using a supporting electrolyte solution such as NaCl and scanning the electrode at fixed scan rate. Afterwards, the double layer capacitance (*C_dl_*) could be calculated using the equation *C_dl_* = Δ*I*/2ν, where Δ*I* is the difference in current between positive and negative potential cycles, and ν is the scan rate [69]. By assuming a specific capacitance (*C_S_*) of the electrolyte solution at the electrode interface, the electroactive area (*A_EA_*) could be estimated by applying *A_EA_* = *C_dl_*/*C_s_* [70]. Moreover, the real surface area can be obtained by scanning the electrode in 0.5 M H_2_SO_4_ solution between −0.3 V and 1.7 V vs. SCE at 100 mV s^−1^. During the potential cycling of PG in H_2_SO_4_ solution, anodic peaks between 1.1 and 1.4 V vs. SCE can be observed due to oxygen layer formation at the electrode interface (notably, O_2_ is assumed to form a monoatomic layer before O_2_ evolution with a one-to-one correspondence with the metal atoms on the electrode surface). Furthermore, a well-defined cathodic peak at 0.9 V can be observed due to the reduction of the gold oxide layer. Afterwards, the cathodic peak can be integrated to obtain the underlined area, which corresponds to the measured charge (*Q_mes_*). The real surface area (*R_SA_*) can be estimated, assuming that a specific charge (*Q_sp_*) of 390 μC cm^−2^ is required for gold oxide reduction and considering *R_SA_* = *Q_mes_*/*Q_sp_* [71]. Additionally, the measurements of double-layer capacitance resulted in being effective for the real surface area estimation. Finally, the composition of the electrodes can be analyzed using different spectroscopic methods such as X-ray photoelectron spectroscopy (XPS) and energy dispersive X-ray spectroscopy (EDX). PG electrodes functionalized with biomolecules at relatively low concentrations have been characterized by using XPS [72,73].

## 4. Porous Gold (PG) Features for Enzyme-Based Bioelectrochemical Applications

PG electrodes possess four main features for bioelectrochemical applications:

*(1) Enhanced enzyme layer stability*: Enzymes that are confined within the pores show higher stability when exposed to high temperatures and organic solvents. Xu and coworkers reported that after incubation at 50 °C for 2 h, free laccase retained only 6% of its initial activity while laccase immobilized onto PG retained almost 60% [72,74]. A similar behavior has been also reported for xylanase and lipase immobilized on PG [73]. The lipase-PG modified electrode retained 81% of its initial activity after treatment with ethyl acetate;

*(2) Increased rate of electron transfer process*: The high content of low index crystalline faces in inner surface of the pores can enhance the rate of electron transfer processes (e.g., glucose, hydrogen peroxide, nitrite, etc.) [6,21,38];

*(3) Enhanced stability of self-assembled monolayers (SAMs) for an efficient binding of redox enzymes*: SAMs exhibit an enhanced stability onto PG modified electrodes compared to planar gold electrodes mainly due to the presence of defective sites, lattice strain and residual silver on the ligament surface. This has been confirmed by performing the electrochemical SAMs desorption in alkaline media [75]. For example, Xiao et al. reported that 3,3′-dithiodipropionic acid (DTDPA), 6-mercaptohexanoic acid (MHA) and 11-mercaptoundecanoic acid (MUA) SAMs were electrochemically desorbed from dealloyed PG. In all cases, this phenomenon showed reduction peaks that are significantly higher than those for planar gold, respectively. Moreover, Hakamada and his coworkers reported that the electrochemical desorption of 4-aminothiophenol occurred at potential of −1.15 V vs. SCE, [76,77], probably because of the heterogeneity of Au-S binding sites available onto the PG films modified electrode [61]. Notably, the binding interactions Au-S are occurring either at basal planes and stepped surfaces, thus requiring a more negative potential for the desorption, as confirmed from voltammetric data available in the literature [78]. The enhanced stability of SAMs onto PG modified electrodes allowed to achieve an efficient binding of redox enzymes for biosensors and EFCs purposes [77];

*(4) Increased antifouling properties:* PG can hinder the access of proteins from accessing the internal pores, reducing the effects of biofouling. This feature plays a key role while performing amperometric measurements in bloods or other body fluids. For example, the initial electrochemical response of [Fe(CN)_6_]^3−^ decreased by 50% in the presence of 2 mg mL^−1^ of bovine serum albumin (BSA) in 3 min on planar gold, while in 12 min on macroporous gold and 38 min on hierarchical gold. Conversely, the current decreased by ca. 12% after 60 min for dealloyed nano-PG electrodes, indicative of a significant resistance to fouling [79,80].

## 5. Porous Gold (PG) Enzyme-Based Electrodes

### 5.1. Dehydrogenases

**Figure 5:**
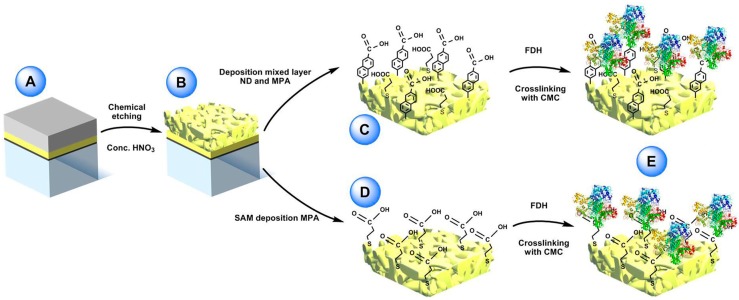
(**A**) Sputtered glass sheet with layers of titanium, pure gold and gold/silver alloy (bottom to top). (**B**) nano-PG electrode surface post-dealloying by concentrated nitric acid. (**C**) Electrochemical reduction of 6-amino-2-Naphthoic diazonium salt (ND) on the nano-PG surface utilizing a single scan and subsequent filling of the void spaces with 3-mercaptopropionic acid (MPA). (**D**) Preparation of mercaptopropionic acid (MPA) SAM on nano-PG substrate by immersion over night at 4 °C. (**E**) After adsorption of FDH on the two modified electrodes the enzyme was crosslinked using *N*-cyclohexyl-*N*’-(2-morpholinoethyl) carbodiimide metho-*p*-toluenesulfonate (CMC). Reproduced with permission from [84]. Copyright Wiley, 2017.

Recently, one- and multicofactor dehydrogenases like flavin adenine dinucleotide (FAD), flavin mononucleotide (FMN) and pyrroloquinoline quinone (PQQ) dependent dehydrogenases, containing sometimes an additional heme-based electron transfer subunit, were demonstrated that can undergo direct electron transfer (DET) at several different electrode platforms [81,82,83]. In particular, Siepenkoetter and his coworkers reported on the covalent immobilization of fructose dehydrogenase (FDH) onto nano-PG electrode, as shown in Figure 5 [84].

FDH is a heterotrimeric membrane-bound enzyme complex, consisting of three subunits, especially subunit I (DH_FDH_), which is the catalytic domain where D-fructose is involved in a 2H^+^/2e^−^ oxidation to produce 5-dehydro-d-fructose, and subunit II (CYT_FDH_), which acts as a built-in electron transfer unit between DH_FDH_ and the electrode surface [85,86,87]. In this paper, nano-PG was reported to be prepared through chemical dealloying of silver-gold alloy by using different temperatures and dealloying times in order to obtain different pore size. Nano-PG electrodes with average pore sizes ranging from 9 to 62 nm have been studied [88]. Indeed, the nano-PG electrode with an average pore size of 42 nm produced the highest current densities. This platform was further used to develop a DET biosensor for the detection of fructose exhibiting a sensitivity of 3.7 ± 0.2 µA cm^−2^ mM^−1^, a limit of detection of 1.2 µM and an increased high thermal (up to 35 °C) and storage stability (40% of initial activity retained after 6 days) mainly due to the porous structure protecting the immobilized enzyme.

In our previous paper, we reported a self-templated electrodeposition of highly-PG obtained with a two-step method, as mentioned above [52]. The so nanostructured electrode was further modified with different self-assembled monolayers owing different functional groups, namely –COOH (4-mercaptobenzoic acid), –OH (4-mercaptophenol) and –NH_2_ (4-aminothiophenol) in order to produce differently charged electrode surface for an effective immobilization of FDH through electrostatically driven adsorption. The electrode modified with 4-mercaptophenol exhibited the largest catalytic current density (about 1000 μA cm^−2^ in the presence of 10 mM fructose) due to the presence of –OH groups on the electrode surface, which stabilize and orientate the enzyme layer on the electrode surface. The so modified electrode displayed excellent analytical features such as a linear range between 0.05 and 5 mM, a sensitivity of 175 ± 15 μA cm^−2^ mM^−1^, a detection limit of 0.3 µM, an increased high thermal (up to 35 °C) and an extraordinary storage stability (90% retained activity over 90 days). In this case, the protection of the enzyme layer is ensured from the large pores (almost 10 µm in diameter) and from their nanostructures where the high number of enzyme molecules is effectively retained.

However, we should consider several examples where dehydrogenases have been immobilized onto PG base electrodes in a mediated electron transfer (MET) architecture. In this regard, Ben-Ali et al. reported the development of a novel electrochemical method for the deposition of highly ordered microporous gold nanostructure by using colloidal lattice as templates because they can be produced with an almost perfect arrangement [89]. Afterwards, the colloidal lattice is removed by using an appropriate solvent, which resulted in a really ordered microporous structure able to support the mediated electron transfer of nicotinamide adenine dinucleotide (NAD)-dependent glucose dehydrogenase (GDH) [90]. The electrode surface was further modified by using the layer-by-layer method alternating the mediator, the NAD^+^ cofactor and the enzyme stabilized from the Nafion^®^ membrane. It should also be mentioned the presence of an intermediate layer of Ca^2+^ between the mediator and NAD^+^, which is well known to enhance the kinetics of the electron transfer between NAD^+^ and the mediator. This supramolecular electrode architecture showed great electrocatalytic activity towards glucose oxidation at relatively low overpotential (almost −100 mV vs. SCE).

In a similar approach, Szamocki and his coworkers reported the immobilization of NAD-dependent GDH onto highly ordered macroporous gold electrode modified with (4-carboxy-2,5,7-trinitro-9-fluorenylidene)malononitrile (TNF) and NAD^+^. The enzyme was cross-linked by using glutaraldehyde. GDH/NAD^+^/TNF/PG electrode displayed great electrocatalytic cyclic voltammograms in presence of glucose [91].

Moreover, Xiao et al. reported the development of a symmetric supercapacitor/biofuel cell based on the electrodeposition of a silver/gold alloy further chemically dealloyed. This PG electrode preparation method has been already deeply discussed in the previous sections. In this study, they were able to support the MET of flavin adenine dinucleotide (FAD)-dependent GDH through the electrodeposited poly(3,4-ethylenedioxythiophene) (PEDOT) and the redox polymer [Os(2,2′-bipyridine)_2_(polyvinylimidazole)_10_Cl]^+/2+^(Os(bpy)_2_PVI). The same electrode construction method has been employed for the immobilization of the cathodic enzyme, namely bilirubin oxidase (BO*x*). This hybrid device allowed us to store in the supercapacitor the energy yielded by the biofuel cell with an effective delivery of a high power pulse. Notably, it released a pulse current density of 2 mA cm^−2^, which correspond to an instant maximum power density of 609 μW cm^−2^ (468 times higher than that of the EFC) [92].

Moreover, Salaj-Kosla et al. reported the immobilization of cellobiose dehydrogenase (CDH) [93,94] onto a similarly prepared PG electrode [95,96]. The enzyme was covalently cross-linked to an osmium-based redox polymer supporting its MET [97,98]. The so modified electrode showed great catalytic current towards lactose oxidation and an exceptionally poor operating stability loosing 70% of the osmium redox polymer initially immobilized onto the electrode.

**Figure 6:**
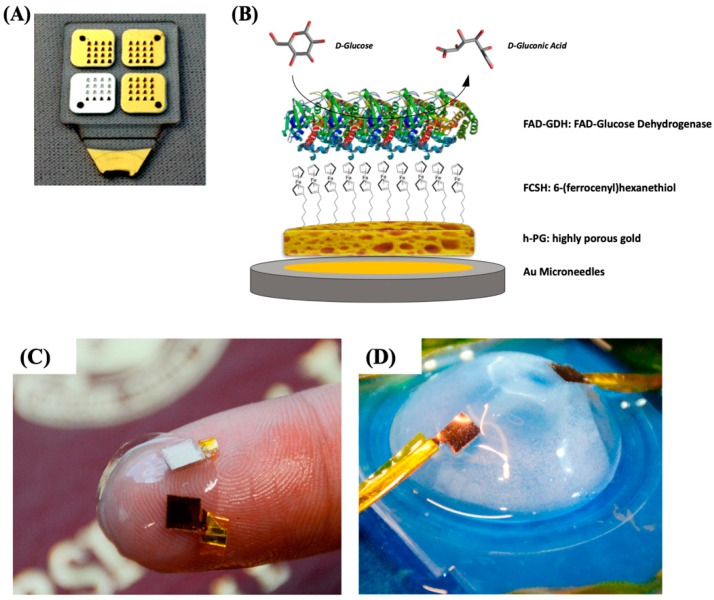
(**A**) Photo of four 4 × 4 microneedle arrays metallized with gold and silver and (**B**) schematic representation of the highly porous gold (h-PG)/6-(ferrocenyl)hexanethiol (FcSH)/flavin adenine dinucleotide glucose dehydrogenase (FAD-GDH)/Au microneedle-based glucose biosensor. Photograph of the contact lens encapsulated enzymatic fuel cell (EFC; **C**) and testing setup (**D**). Figure 6A,B are reproduced with permission from [100]. Copyright MDPI, 2019. Figure 6C,D are reproduced with permission from [105]. Copyright American Chemical Society, 2018.

In our previous paper, we reported the self-templated electrodeposition of PG gold onto microneedle-based electrodes [99] developing a minimally invasive biosensor for the detection of glucose based on MET of FAD-dependent GDH [100,101]. The highly porous gold surface of the microneedles was further modified by using 6-(ferrocenyl)hexanethiol (FcSH) as a redox mediator and drop-casting the FAD-GDH enzyme solution, as schematically reported in Figure 6A,B. The microneedle-based FcSH/FAD-GDH biosensor showed an extended linear range (0.1–10 mM), high sensitivity (50.86 µA cm^−2^ mM^−1^) and good storage stability (20% signal loss after 30 days).

### 5.2. Oxidases

The group of oxidases is another family of redox enzymes that has been successfully immobilized onto PG based electrodes. Oxidases have been widely employed for the development of first generation (oxidation current is mainly related with the oxidation/reduction of H_2_O_2_ at the electrode) and second generation biosensors (the electrons are shuttled from a mediator to the electrode) [1,102].

Du Toit and coworkers [50,103] reported on the potentiostatic self-templated electrodeposition of highly PG layer by using two steps: (i) small hydrogen bubbling by applying −0.7 V vs. SCE and (ii) a vigorous hydrogen bubbling by applying −4 V vs. SCE. Afterwards, the authors explored two different immobilization methods, one based on the electrodeposition of glucose oxidase (GO*x*) by cycling the electrode between 0.42 and 0.6 V vs. SCE, and the other one by simple adsorption of the enzyme onto the electrode surface. The first method resulted in a more intimate contact between the redox enzyme and the pore internal surface enhancing by approximately 6 times the glucose oxidation current compared to the electrode obtained through enzyme adsorption. The so modified electrode has been reported either as first generation biosensor for glucose detection and as a bioanode of EFC.

Moreover, Szamocky et al. [104] reported on GO*x* immobilization onto a highly ordered macroporous electrode by incorporating the enzyme into an electrodeposition paint (EDP). The electrodeposition paint is a copolymer of acrylic acid and various acrylates (water soluble in the original form). By applying a sufficiently positive voltage to oxidize water, the pH decreases locally, therefore the acrylic acid gets protonated forming a thin layer on the electrode and incorporating GO*x*. The so prepared electrode showed great catalytic properties towards glucose oxidation, which is detected through H_2_O_2_ electrochemical oxidation at the electrode surface.

Furthermore, Sanzò et al. reported the immobilization of GOx onto a one-step self-templated PG electrode for the development of a first generation glucose biosensor. GO*x* was easily immobilized through covalent cross-linking with glutaraldehyde. The so modified electrode showed a higher sensitivity of 48.3 ± 0.9 μA cm^−2^ mM^−1^ at 0.45 V vs. SCE compared to a value of 24.6 ± 1.3 μA cm^−2^ mM^−1^ at 0.70 V vs. SCE obtained with bare Au electrodes [51].

Xiao et al. [105] reported the immobilization of lactate oxidase (LO*x*) onto nano-PG electrode (obtained through electrodeposition and successive chemical dealloying) for the development of EFC onto contact lenses. After the preliminary step, the PG electrode was further modified with an osmium redox polymer in order to promote the MET of LO*x*, while BO*x* was immobilized in direct electron transfer [106]. Once prepared, the electrodes were placed between two contact lenses in order to obtain a flexible EFC, as displayed in Figure 6C,D. The EFC showed a maximum power density of 1.7 ± 0.1 μW cm^−2^ and an open-circuit voltage of 380 ± 28 mV in air-equilibrated artificial tears solution (containing approximately 3 mM lactate). A good EFC operational stability could be observed retaining 20% of its initial power output after 5.5 h maybe due to the protective effect of porous nanostructured electrodes.

### 5.3. Heme Proteins

Cytochrome c (*cyt c*) has been widely used as a model protein that can undergo direct electron transfer onto self-assembled monolayer (SAM) modified electrode [107]. *Cyt c* electrochemical behavior is greatly affected by the topography of gold electrodes (enzyme immobilized through easy drop-casting). In particular, it was demonstrated that highly rough gold surfaces could result in SAMs containing multiple defects, ensuring an increased *cyt c* surface coverage. A PG electrode modified with *cyt c* covalently immobilized onto a mixed SAM showed a well-defined and almost symmetric voltammogram (peak-to-peak separation Δ*E_p_* = 18 mV). The surface coverage of active *cyt c* onto the modified electrode was approximately 11 times higher than that achieved at a planar Au surface. The so prepared electrode showed an extremely high operating stability with no decreases in the voltammetric response under continuous potential cycling (30 scans) in an aqueous buffer [108].

### 5.4. Multicopper Oxidases (MCOs)

Multicopper oxidases group (MCOs, e.g., laccase and bilirubin oxidase (BOx)) includes many enzymes able to reduce dioxygen to water (four electrons) with the contemporary one-electron oxidation of an electron donor (e.g., catechol, 2,2’-azino-bis(3-ethylbenzothiazoline-6-sulfonic acid) (ABTS), etc.) [109]. MCOs modified electrodes have been mainly used as biocathodes of EFCs [110]. MCOs contain four copper atoms: the T1 copper site acts as an electron acceptor (DET configuration: electrons donated from the electrode and MET configuration: electrons donated from a diffusive or immobilized mediator) while O_2_ is reduced to H_2_O at the T2/T3 trinuclear copper cluster [111,112].

**Figure 7:**
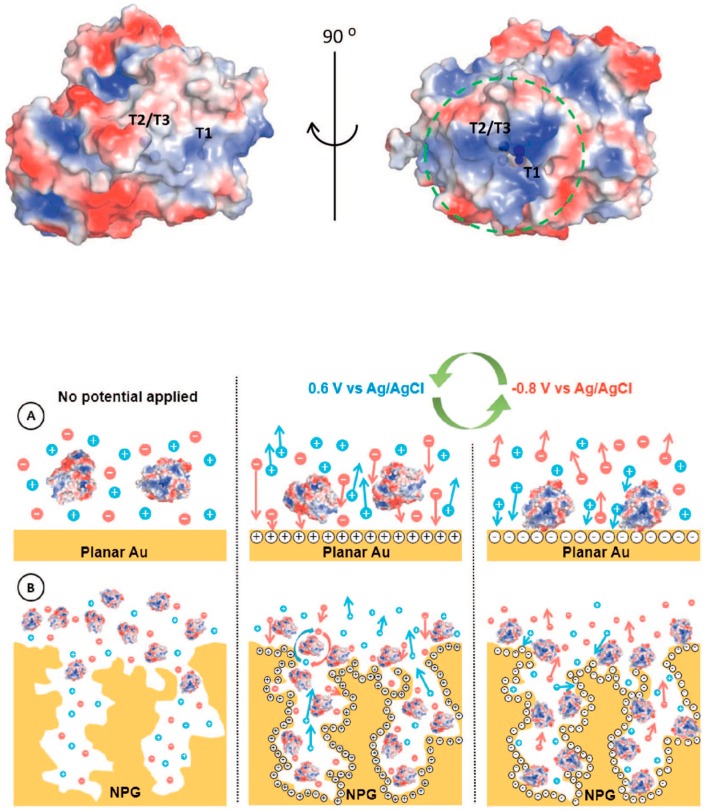
Surface charge distribution pattern of (PBD 2XLL) *Myrothecium verrucaria* BO*x* (blue and red colors symbolize positive and negative charges, respectively). Potential pulse-assisted immobilization approach at (**A**) planar gold and (**B**) nano-PG electrodes. The green arrows indicate the changes in the potential pulses of below and above pzc values. Reproduced with permission from [113]. Copyright Elsevier Ltd., 2018.

In this regard, Lopez and coworkers reported the immobilization of BO*x* onto nano-PG electrodes by using a potential pulse method [113]. The potentials applied were selected based on the potential of zero charge (pzc), which resulted to be different for a polycrystalline gold electrode and PG electrodes with different morphology, as reported in Figure 7. Indeed, the latter resulted to be the critical factor for the determination of potential needed for the enzyme deposition. This method allowed a selective orientation of BO*x* with the T1 site toward the electrode in order to be able to accept electrons directly from the electrode (DET) [114]. Thus, the high values of current density related to dioxygen reduction could mainly be attributed to the enhanced enzyme surface coverage and to the efficient enzyme orientation.

In a similar approach, Salaj-Kosla et al. reported the immobilization of high potential laccase, namely *Thrametes hirsuta* laccase, onto nano-PG electrode [115]. In this case, the immobilization was initially performed through adsorption of the enzyme into the porous structure, resulting in poor catalytic performance and a continuous leakage of the enzyme from the electrode. Thus, the electrode surface was later stabilized by using epoxy-based polymers able to cross-link the enzyme into the porous structure. However, the authors investigated also the resistivity of the enzyme modified electrode towards two different inhibitors, namely fluoride and chloride. It should be mentioned that fluoride acts as a non-competitive inhibitor by binding to the T2/T3 copper cluster, blocking the ET pathway from the T1 site to the T2/T3 cluster. This results in the absence of the electrocatalytic wave typical for dioxygen reduction, even with a minimal presence of fluoride. Conversely, chloride is considered a competitive inhibitor, which hinders the electron pathway between the electron donors and the T1 site (DET: electrodes; MET: diffusive or immobilized mediator). In the latter case, the nano-PG electrode was able to retain 50% of its initial activity after the addition of 150 mM of Cl^−^, probably due to the intimate contact between the enzyme and the pore surface [116]. Moreover, the electrode showed also a good thermal stability gaining 40% of activity by increasing the temperature up to 37 °C. The last feature of this electrode opens some future avenue for its employment in implantable devices. Moreover, the electrodes reported above have been successfully used to develop glucose/O_2_ or lactate/O_2_ EFCs [2,105,110].

## 6. Conclusions and Future Perspectives

PG electrodes have been proven to be a promising support for enzyme immobilization improving their response and stability towards multiple enzyme-based bioelectrochemical applications (e.g., biosensors and biofuel cells). Notably, PG modified electrodes exhibit four main advantages towards the development of enzyme-based devices, namely increasing the stability of the immobilized enzyme, enhanced electron transfer reaction rate, increasing the stability of self-assembled monolayers (SAMs) and hindering of surface biofouling. In particular, it was demonstrated that PG layers could increase the stability of enzyme layers when exposed to high temperatures and organic solvents. Moreover, PG electrodes enhanced the stability of SAMs allowing a stable covalent linking of redox enzymes. Finally, it was clearly demonstrated the possibility to drastically increase the ET rate constants of immobilized redox enzymes as well as good anti-biofouling properties.

In the second part, the most recent findings about enzyme immobilization onto PG modified electrodes have been carefully revised. PQQ-/FAD- dependent dehydrogenases as well as multicopper oxidases (MCOs) able to give DET exhibited an efficient enzyme orientation onto the electrode surface in order to enhance the ET rate. This was successfully exploited to construct really sensitive biosensors and efficient biofuel cells. However, also oxidases and other dehydrogenases working in MET showed great performance in terms of the sensing platform sensitivity, stability and limit of detection.

Overall, the porous nature of the electrode support allowed for the immobilization of higher amounts of enzyme than planar gold, which might not be optimal for very deep pores where diffusion of enzyme substrate into the pores can become rate limiting. This limitation could eventually be overtaken by using of flow systems, enhancing the substrate transport.

In a future sight, PG electrodes can be potentially deposited on plastic supports to realize flexible devices, enabling the development of thin flexible polymer-based electronic devices (e.g., smart contact lenses, implantable biosensors, smart drug delivery devices, etc.) for biomedical applications [117,118].
